# A Nanoscaffolded Spike-RBD Vaccine Provides Protection against SARS-CoV-2 with Minimal Anti-Scaffold Response

**DOI:** 10.3390/vaccines9050431

**Published:** 2021-04-27

**Authors:** Duško Lainšček, Tina Fink, Vida Forstnerič, Iva Hafner-Bratkovič, Sara Orehek, Žiga Strmšek, Mateja Manček-Keber, Peter Pečan, Hana Esih, Špela Malenšek, Jana Aupič, Petra Dekleva, Tjaša Plaper, Sara Vidmar, Lucija Kadunc, Mojca Benčina, Neža Omersa, Gregor Anderluh, Florence Pojer, Kelvin Lau, David Hacker, Bruno E. Correia, David Peterhoff, Ralf Wagner, Valter Bergant, Alexander Herrmann, Andreas Pichlmair, Roman Jerala

**Affiliations:** 1 EN-FIST, Centre of Excellence, 1000 Ljubljana, Slovenia; dusko.lainscek@ki.si (D.L.); iva.hafner@ki.si (I.H.-B.); mateja.mancek@ki.si (M.M.-K.); mojca.bencina@ki.si (M.B.); 2 Department of Synthetic Biology and Immunology, National Institute of Chemistry, 1000 Ljubljana, Slovenia; tina.fink@ki.si (T.F.); vida.forstneric@ki.si (V.F.); sara.orehek@ki.si (S.O.); ziga.strmsek@ki.si (Ž.S.); peter.pecan@ki.si (P.P.); hana.esih@ki.si (H.E.); spela.malensek@ki.si (Š.M.); jana.aupic@ki.si (J.A.); petra.dekleva@ki.si (P.D.); tjasa.plaper@ki.si (T.P.); sara.vidmar@ki.si (S.V.); lucija.kadunc@ki.si (L.K.); 3 Graduate School of Biomedicine, University of Ljubljana, 1000 Ljubljana, Slovenia; 4 Department of Molecular Biology and Nanobiotechnology, National Institute of Chemistry, 1000 Ljubljana, Slovenia; neza.omersa@ki.si (N.O.); gregor.anderluh@ki.si (G.A.); 5 Protein Production and Structure Core Facility PTPSP- EPFL SV PTECH PTPSP, 1015 Lausanne, Switzerland; florence.pojer@epfl.ch (F.P.); kelvin.lau@epfl.ch (K.L.); david.hacker@epfl.ch (D.H.); bruno.correia@epfl.ch (B.E.C.); 6 Molecular Microbiology (Virology), Institute of Medical Microbiology and Hygiene, University of Regensburg, 93053 Regensburg, Germany; david.peterhoff@klinik.uni-regensburg.de (D.P.); ralf.wagner@klinik.uni-regensburg.de (R.W.); 7 Institute of Clinical Microbiology and Hygiene, University Hospital Regensburg, 93053 Regensburg, Germany; 8 Immunopathology of Virus Infections Laboratory, Institute of Virology, Technical University of Munich, 81675 Munich, Germany; valter.bergant@tum.de (V.B.); alexander.herrmann@tum.de (A.H.); andreas.pichlmair@tum.de (A.P.); 9 German Center for Infection Research (DZIF), Munich Partner Site, 38124 Braunschweig, Germany

**Keywords:** SARS-CoV-2, RBD-bann, nano-scaffolding domains, vaccine, T-cell response

## Abstract

The response of the adaptive immune system is augmented by multimeric presentation of a specific antigen, resembling viral particles. Several vaccines have been designed based on natural or designed protein scaffolds, which exhibited a potent adaptive immune response to antigens; however, antibodies are also generated against the scaffold, which may impair subsequent vaccination. In order to compare polypeptide scaffolds of different size and oligomerization state with respect to their efficiency, including anti-scaffold immunity, we compared several strategies of presentation of the RBD domain of the SARS-CoV-2 spike protein, an antigen aiming to generate neutralizing antibodies. A comparison of several genetic fusions of RBD to different nanoscaffolding domains (foldon, ferritin, lumazine synthase, and β-annulus peptide) delivered as DNA plasmids demonstrated a strongly augmented immune response, with high titers of neutralizing antibodies and a robust T-cell response in mice. Antibody titers and virus neutralization were most potently enhanced by fusion to the small β-annulus peptide scaffold, which itself triggered a minimal response in contrast to larger scaffolds. The β-annulus fused RBD protein increased residence in lymph nodes and triggered the most potent viral neutralization in immunization by a recombinant protein. Results of the study support the use of a nanoscaffolding platform using the β-annulus peptide for vaccine design.

## 1. Introduction

COVID-19 is a pandemic viral disease caused by SARS-CoV-2 that emerged in 2019 and has infected tens of millions of people across the world, with over one million casualties. Massive vaccination could stop the waves of infection that continue to spread throughout the world. Different vaccination platforms for the presentation of viral proteins have been tested, including inactivated viruses, mRNA, and adenoviral delivery of spike protein-coding nucleic acids and recombinant proteins [1,2,3,4,5,6,7]. The advantages of DNA plasmid delivery, including rapid adaptation for new targets, cost-effective production, and stability at ambient temperatures, provide a potentially attractive vaccine platform, with several veterinary DNA plasmid vaccines already approved [8,9]. The majority of vaccines are based on a trimeric full-length spike protein or its stabilized derivatives, through which the virus attaches to the host cell receptor ACE2 [10]. In this case, antibodies against the surface-exposed epitopes of the spike protein are generated, where some of them may not block recognition of the ACE2 receptor and viral entry and might even facilitate antibody-dependent enhancement (ADE), as suggested before for SARS CoV and MERS CoV [11,12,13]. Immune response against the receptor binding domain (RBD) of the spike protein induces formation of neutralizing antibodies, [2,7], and monoclonal antibodies targeting RBD have demonstrated effectiveness [14,15]. Viral proteins are typically presented to the immune system in the form of nanoparticles, which present tens of copies of viral proteins on their surface. Particles that present multiple copies of the antigen are more immunogenic than monomeric proteins due to the clustering of B cell receptors, increased avidity of multimeric proteins, and augmented retention of nanoparticles above 20 nm in the lymph nodes [16,17,18,19]. Larger nanoparticles are retained longer inside lymph node follicles and presented at the dendrites of follicular dendritic cells [20]. To mimic natural infection and induce an optimal host immune response, immunogenic domains have been attached to scaffolds, such as capsid proteins of viruses (Qβ, HPV, JCV, HBcAg, cowpea chlorotic mottle virus [21]); proteins such as ferritin, lumazine synthase, and encapsulin [18,22,23,24,25]; *de novo* designed protein or DNA cages [26,27,28,29,30,31]; or peptide tags with high aggregation propensity.

Here, we compared several strategies of presentation of the RBD domain of the SARS-CoV-2 spike protein on scaffolded particles with different stoichiometries. The RBD assemblies were genetically encoded and tested on animals in the form of a DNA plasmid vaccine encoding secreted fusion proteins. We show that RBD fused to scaffolding domains strongly increased the titer of S-protein- and RBD-specific antibodies in comparison to a vaccine encoding RBD alone. Antibodies produced in response to RBD-scaffolded fusions recognized the spike protein, neutralized binding of the spike protein to the ACE2 receptor, and induced a robust T-cell response, confirming the assistance of higher-order structures in efficient induction of the immune response. The β-annulus peptide has been previously shown to form large soluble nanoparticles [32]. Interestingly, a potent antibody response was obtained when fusing RBD to the β-annulus peptide (RBD-bann), including a high level of neutralization and significant protection in a surrogate infection assay, a robust T-cell response, and prolonged retention in lymph nodes in vivo. An important issue of the scaffolding strategy is that antibodies may also be targeted against the scaffolding domain or a delivery vector, which could impair the efficiency of subsequent immunizations with the same vaccine type [33,34]. We surmised that the implementation of small hypoimmunogenic scaffolding domains could represent an efficient vaccine platform. Vaccine variants in which the scaffold represented the smallest fraction generated a very low level of scaffold-directed antibodies even after a booster dose in mice. Importantly, scaffolding based on the small β-annulus peptide, which triggered the most potent immune response, induced almost no scaffold-directed antibodies in contrast to other scaffolds analyzed in the study, such as ferritin and lumazine synthase. Vaccine protection against infection was demonstrated in a novel, safe, and easy-to-implement mouse model, allowing in vivo experiments to be performed in a BSL2 set-up. Further, this type of nanoscaffolding strategy could be implemented in DNA, mRNA, viral or isolated protein-based platforms, or combinations thereof in well-considered prime-boost regimens. Indeed, immunization with isolated proteins demonstrated a strong potentiation of the response to the β-annulus-scaffolded variant, which can be explained by augmented retention in the lymph nodes. The results obtained in this study represent groundwork for new vaccine design platform.

## 2. Materials and Methods

### 2.1. Modeling of the Designed RBD-Scaffolded Protein Cages

The molecular model structures of designed nanovaccines were prepared by performing homology modeling with Modeller (version 9.23, San Francisco, CA, USA) [35]. The structures PDB ID: 6VW and PDBID: 6VSB were used as templates for the RBD. For the RBD-ferritin construct, composed of 24 domains, PDB ID: 3EGM was used as a template for the ferritin cage. RBD-foldon-RBD was modeled as a trimer based on the template PDB ID: 1RFO. For RBD-bann, homology modeling was used to first construct a model of the trimeric subunit based on the structure of the tomato bushy stunt virus (TBSV) (PDB ID: 2TBV). Twenty trimeric subunits were then assembled into an RBD-decorated protein cage by employing icosahedral symmetry characteristic for TBSV, resulting in a 60-mer with a diameter of approximately 26 nm. The model of the design RBD-AaLS, composed from 60 subunits, was built by employing PDBID: 1HQK as a template for the lumazine synthase scaffold.

### 2.2. Preparation of DNA Constructs

DNA constructs were prepared using conventional methods, based either on synthetic DNA (Twist, San Francisco, CA, USA or IDT, Coralville, Iowa, United States) or purchased commercially (Genscript, Piscataway, NJ, USA), or clones from plasmids with viral proteins generously provided by Prof. Nevan Krogan, UCSF [36]. The construct encoding the prefusion ectodomain of the SARS-CoV-2 spike protein was a generous gift from Prof. Jason McLellan, University of Texas, Austin [37]. The RBD domain of the spike protein (residues R319 to S591) was codon-optimized for *H. sapiens* and synthesized (Genscript, Piscataway, NJ, USA) into pcDNA3.1 (+) with a human pregnancy-specific glycoprotein 1 signal peptide at the N-terminus and a 3C-protease cleavage site followed by a His-tag at the C-terminus. ACE-2 (hsACE-2, residues S19 to D615) was codon optimized for *H. sapiens* and cloned into the pTwist_CMV_BetaGlobin_WPRE_Neo (Twist Biosciences, San Francisco, CA, USA). It was preceded with a human pregnancy-specific glycoprotein 1 signal peptide and C-terminally tagged with a 3C protease cleavage site, twin-strep tag, and 10x His tag. The RBD domain of the spike protein encompassing residues P330 to K521 was fused with polypeptide scaffolds and inserted into a pcDNA3.1 (+) vector with a CD45 signal peptide at the N-terminus. Fusions of RBD to the β-annulus-scaffold peptide from the tomato bushy stunt virus [32] (RBD-bann), a chimeric fusion of the bullfrog (Rana catesbeiana) and Helicobacter pylori ferritin [38], (RBD-ferritin), lumazine synthase from *Aequifex aeolicus* [39], (RBD-AaLS) and foldon from T4 bacteriophage fibiritin [40] (RBD-foldon-RBD) were codon optimized for expression in mammalian cells. Non-tagged versions of the RBD-scaffold constructs were prepared in parallel for immunization purposes.

### 2.3. Cell Culture

The human embryonic kidney (HEK) 293, HEK293T (ATCC) cell line and mouse NIH-3T3 cell (ATCC, Manassas, VA, USA) line were cultured in DMEM (Invitrogen, Waltham, MA, USA) supplemented with 10% FBS (BioWhittaker) at 37 °C in a 5% CO_2_ environment. ExpiCHO cells (Thermo Fisher, Waltham, MA, USA) were cultured in ProCHO5 medium (Lonza) and incubated with agitation at 31 °C and 4.5% CO_2_. HEK293E cells were cultured in an EX-CELL 293 serum-free medium (Sigma, St. Louis, MO, USA) and incubated with agitation at 37 °C and 4.5% CO_2_. Expi293F cells (Thermo Fisher, Waltham, MA, USA) were cultivated in an Expi293™ Expression Medium (Thermo Fisher, Waltham, MA, USA) at 37 °C and 8% CO_2_ on an orbital shaking platform with the shaking speed based on shaking diameter and flask size.

### 2.4. Recombinant Viral Proteins

The prefusion ectodomain of the SARS-CoV-2 spike protein was transiently transfected into suspension-adapted ExpiCHO cells (Thermo Fisher) with PEI MAX (Polysciences, Warrington, PA, USA) in a ProCHO5 medium (Lonza, Basel, Switzerland). After 1 h, dimethyl sulfoxide (DMSO; AppliChem) was added to 2% (*v*/*v*). Incubation with agitation was performed at 31 °C and 4.5% CO_2_ for five days. The clarified supernatant was purified via a Strep-Tactin column (IBA Lifesciences, Göttingen, Germany) and dialyzed into a PBS buffer. The average yield for the spike protein was 15 mg/L culture. The RBD domain of the spike protein (residues R319 to S591 of Spike) was transiently transfected into suspension-adapted ExpiCHO cells (Thermo Fisher, Waltham, MA, USA) with PEI MAX (Polysciences) in a ProCHO5 medium (Lonza). After 1 h, dimethyl sulfoxide (DMSO; AppliChem) was added to 2% (*v*/*v*). Incubation with agitation was performed at 31 °C and 4.5% CO_2_ for six days. The clarified supernatant was purified in two steps—via a Fastback Ni^2+^ Advance resin (Protein Ark) followed by a Superdex 200 16/600 column (GE Healthcare, Chicago, IL, USA) and finally dialyzed into a PBS buffer. The average yield for RBD-His was around 25 mg/L culture. ACE-2 protein (hsACE-2, residues S19 to D615) was transiently transfected into HEK293E cells (Thermo Fisher, Waltham, MA, USA) with PEI MAX (Polysciences, Warrington, PA, USA) in an EX-CELL 293 serum-free medium (Sigma), supplemented with 3.75 mM valproic acid. Incubation with agitation was performed at 37 °C and 4.5% CO_2_ for eight days. The clarified supernatant was purified in three steps—via a Fastback Ni^2+^ Advance resin (Protein Ark, Sheffield, UK) and a Strep-Tactin XT column (IBA Lifesciences, Göttingen, Germany), followed by a Superdex 200 16/600 column (GE Healthcare, Chicago, IL, USA) and finally dialyzed into a PBS buffer. The average yield for hsACE-2 was 12 mg/L culture.

### 2.5. Production and Characterization of RBD Variants

Expi293F (Thermo Fisher) cells were transiently transfected with the ExpiFectamine™ 293 Transfection Kit (Thermo Fisher) according to the manufacturer’s recommendations and grown in Expi293™ expression medium (Thermo Fisher). Incubation with agitation was performed at 37 °C and 8% CO_2_ for five days. For proteins containing Histag, cell broth was collected after five days and clarified by centrifugation, followed by overnight dialysis against a NiNTA A buffer (50 mM Tris, 150 mM NaCl, 10 mM imidazole, pH 8) with dialysis tubes and a 6–8 kDa cutoff (Spectrum Laboratories, Rancho Dominguez, CA, USA). Samples were purified using NiNTA resin (Goldbio, St Louis, MO, USA) and eluted with 250 mM imidazole in NiNTA A buffer. For proteins containing strep-tag, cell broth was also collected after five days and clarified by centrifugation, followed by purification, using Strep-trap (Cytiva, Marlborough, MA, USA), according to the manufacturer’s recommendations. After affinity isolation, all samples were dialyzed against 20 mM Tris, 150 mM NaCl, pH 7.5.

### 2.6. ELISA Assay for Binding of Recombinant Proteins to Immobilized Human ACE2

For the ELISA assay, high-binding half-well plates (Greiner, Kremsmünster, Austria) were coated with hsACE2 (100 ng/well) and incubated overnight at 4 °C. Next, plates were washed with 1xPBS + 0.05% Tween20 and blocked for 1 h at RT with 100 μL of ELISA/ELISPOT diluent solution (eBioscience, San Diego, CA, USA). After washing, samples (serial dilutions of recombinant RBD proteins or buffer) were incubated for 2 h at room temperature with 1X ELISPOT Diluent. Then followed the steps of washing, detection with Ab Recombinant Anti-SARS-CoV-2 spike glycoprotein antibody (Abcam; ab273169; diluted 1:2000; 1 h room temperature), and washing again. After the addition of the substrate (TMB solution), the reaction was stopped with 0.16 M sulfuric acid. The multiplate reader SineryMx (BioTek, Winooski, VT, USA) was used to measure absorbance. Absorbance at 620 nm was used for correction and was subtracted from the absorbance at 450 nm.

### 2.7. Surface Plasmon Resonance (SPR) Experiments

The dissociation of RBD and RBD-bann from ACE2 was measured with the SPR instrument Biacore X100 (GE Healthcare, US). All binding experiments were performed at 22 °C with 20 mM Tris, 150 mM NaCl, and pH 7.4 as a running buffer. ACE2 was immobilized on the CM5 sensor chip (Cytiva, Marlborough, MA, USA) via amine coupling following the manufacturer’s recommended procedure. The carboxymethylated dextran surface was activated with a 7-min injection at 5 µL/min of a 1:1 ratio of 0.4 M 1-ethyl-3-(3-dimethylaminopropyl)carbodiimide hydrochloride (EDC)/0.1 M N-hydroxy succinimide (NHS). ACE2 (Genscript, Piscataway, NJ, USA) was diluted to 27 µg/mL in a sodium acetate buffer, pH 4.4, and injected over the activated surface on flow cell 2 for 200–400 s. Flow cell 1 was left untreated to serve as a reference for non-specific binding. After the deactivation of the residual reactive sites on the surface with a 7-min injection of 1 M ethanolamine, pH 8.5, the immobilization rate of ACE2 was 2300–7700 response units. Then, 200 nM RBD and RBD-bann were injected over immobilized ACE2 at a constant flow rate of 30 µL/min for 2 min and left to dissociate for 15 min. Buffers for blank injections were adjusted to match the sample buffer composition. The obtained sensorgrams were processed with the BiaEvaluation software (GE Healthcare, Chicago, IL, USA). The binding of analytes to the reference flow cell and the response of the blank injection were subtracted from the corresponding analyte injection. Kinetic parameters were determined from the processed data by globally fitting *k*_a_ and *k*_d_ to a 1:1 binding model. The natural logarithms of normalized responses were plotted against dissociation time to show the difference between the dissociation of RBD-nanoscaffolds compared to RBD. The experiments were repeated at least six times for each construct, and the results shown are the representative dissociation curves for RBD and RBD-bann.

### 2.8. Batch Dynamic Light Scattering Measurement

The size of the proteins was measured on a ZetasizerNano (Malvern, Worcestershire, UK) at 25 °C using an angle of 173° and a 633-nm laser. Particle size distribution was recorded, and the data were analyzed using the software provided by Malvern.

### 2.9. SEC-MALS Analysis

SEC-MALS analysis was performed with an HPLC system (Waters, Milford, MA, USA), coupled with UV- (Waters), Dawn8+ MALS- (Wyatt, Dernbach, Germany), and RI- (Shodex, Tokyo, Japan) detectors. Samples were filtered using 0.1 μm centrifugal filters (Millipore, Burlington, MA, USA) and injected onto Superdex 200 Increase 10/300 column (Cytiva, Marlborough, MA, USA), previously equilibrated with 20 mM Tris pH 7.5, 150 mM NaCl. Data were analyzed using Astra 7.0.

### 2.10. Western Blot

Proteins were separated by SDS-PAGE in reduced condition and transferred to a Hybond ECL nitrocellulose membrane (GE Healthcare, Chicago, IL, USA). Western blot membranes were incubated with appropriate antibodies. The immunoblots were visualized on G-box (Syngene, Cambridge, UK).

### 2.11. Plasmid Stability Assay

Plasmid coding RBD-bann was incubated at −20 °C, +4 °C, or at room temperature and analyzed after seven days of incubation. The plasmids were analyzed on an agarose gel and transfected into Expi293F cells to determine the expression of the RBD-bann protein. The cells were harvested five days post-transfection and analyzed by a sandwich ELISA assay using anti-RBD antibodies (capture Abs SARS-CoV-2 (2019-nCoV) Spike Antibody (Sinobiological 40150, Beijing, China) 1:500, detection Abs Anti-SARS-CoV-2 spike glycoprotein antibody [H6]-Chimeric (HRP) (Abcam ab273169, Abcam, Cambridge, UK) 1:2000).

### 2.12. Mouse Immunization Studies

To test the immunogenicity of the DNA vaccines, female 8–10-week-old BALB/c OlaHsd mice (Envigo, Desio MB, Italy) were used for immunization protocols. All animal experiments were performed according to the directives of the EU 2010/63 and were approved by the Administration of the Republic of Slovenia for Food Safety, Veterinary, and Plant Protection of the Ministry of Agriculture, Forestry, and Foods, Republic of Slovenia (Permit Number U34401-8/2020/9, U34401-8/2020/15, U34401-12/2020/6). Laboratory animals were housed in IVC cages (Techniplast, Buguggiate, Italy) and fed standard chow (Mucedola, Milano, Italy) and tap water was provided ad libitum. Mice were maintained in a 12–12-h dark–light cycle. All animals used in the study were healthy and accompanied by a health certificate from the animal vendor.

Immunization was carried out under general inhalation (1.8% MAK isoflurane anesthesia (Harvard Apparatus, Holliston, MA, USA)). The immunization protocol was based, if not stated otherwise, on prime vaccination and two boosts, with a two-week interval between vaccinations. Animals were vaccinated with plasmid DNA (RBD, RBD-scaffold, scaffold vectors, empty pcDNA3.1 (+) vector), either naked DNA or coupled with the jetPEI-in vivo transfection reagent (Polyplus Transfection, New York, NY, USA) with the N/P ratio 12. Each animal received a total of 20 μg of the designated plasmid DNA combined with the transfection reagent via intramuscular injection (total volume of 50 μL per animal). For intranasal immunization, plasmid DNA was administered by slowly pipetting the plasmid DNA suspension (plasmid DNA in 0.9% saline solution; 20 μL per mouse) onto the left and right nostril (5 μL suspension at once with 3-s interval). For sublingual immunization, plasmid DNA (20 μg per mouse; approximately 4 μL of plasmid DNA) was pipetted under the tongue of the anesthetized animal. After that, the animal remained anesthetized for an additional 20 min to achieve maximal absorption of the plasmid DNA.

In the stated experiments, the animals received naked plasmid DNA or isolated recombinant proteins (100 μg/animal of designated recombinant protein, coupled with/without squalene adjuvant (50%/animal) AddaVax^TM^ (Invivogen; vac-adx-10, San Diego, CA, USA).

The vaccine was administered using a 30 G needle (Beckton Dickinson, Franklin Lakes, NJ, USA) into *m.tibialis* anterior after appropriate area preparation. One day before each boost, blood was drawn from the lateral tail vain using Microvette 300 (Sarstedt, Newton, NC, USA). Two weeks after the second boost, the experiment was terminated. Final blood was taken, and spleens were harvested from the animals for further analysis. Mouse sera were prepared by centrifugation of blood samples at 3000 RPM for 20 min at 4 °C. In mouse sera, specific mouse antibodies were determined by ELISA in order to analyze the immunogenicity of the designed RBD DNA vaccines. In a different experiment, switch immunization was performed, where the prime and boost were carried out with RBD DNA fused to two different scaffolds; for example, the prime was carried out with bann-RBD and the boost with RBD-foldon-RBD and vice versa.

### 2.13. Analysis of Immune Response on Mice

ELISA tests were performed to determine endpoint titers for designated specific antibodies. High-binding half-well plates (Greiner, Kremsmünster, Austria) were used. Recombinant proteins were coated in a PBS buffer (Gibco, Thermo Fisher Scientific, Waltham, MA, USA) at a concentration of 1.2 mg/mL of protein per well overnight at 4 °C. The plates were washed with PBS + 0.05% Tween20 (PBS-T) using the ELISA plate washer (Tecan, Männedorf, Switzerland) and blocked for 1 h at RT with 100 μL of ELISA/ELISPOT diluent solution (eBioscience). Serial dilutions of sera were added to the plates, where each dilution presented a certain titer value. In the first row, a 1:100 dilution was added, then a three-fold dilution was performed with each row and incubated at 4 °C overnight. Specific secondary antibodies (dilution 1:3000), coupled with HRP, were added to wells. For total IgG determination, goat anti-mouse IgG (H + L)-HRP antibodies (Jackson ImmunoResearch; 115-035-003, West Grove, PA, USA) were used. To determine specific types of anti-RBD antibodies in the mouse sera, goat anti-mouse IgG1-HRP (Abcam; ab97240, Abcam, Cambridge, UK), goat anti-mouse IgG2a heavy chain-HRP (Abcam; ab97245, Abcam, Cambridge, UK), goat anti-mouse IgG2b heavy chain-HRP (Abcam; ab97250, Abcam, Cambridge, UK), goat anti-mouse IgG3 heavy chain-HRP (Abcam; ab97260, Abcam, Cambridge, UK), goat anti-mouse IgM mu chain-HRP (Abcam; ab97230, Abcam, Cambridge, UK), and goat anti-mouse IgA alpha chain-HRP (Abcam; ab97235) were used. EPT was determined as the dilution above the value of the cutoff. The cutoff value was determined from the absorbance data of the control animals (vaccinated with empty vector pcDNA3.1(+)) [41]. For a confidence level of 95%, a value of 2335 was used as the standard deviation multiplier.

### 2.14. VSVΔG* Pseudotyped Virus System and Pseudoviral Neutralization Assay

A pseudovirus system based on the vesicular stomatitis virus [42] was used to determine the virus neutralization capacity of immunized mice sera, antigen-specific cellular immunity, and animal infection studies. Plasmids and the VSVΔG*/G virus were kindly provided by Stefan Pölhmann. Pseudovirus preparation was described previously [10]. Briefly, HEK293T cells were seeded into 6-well plates (9 × 10^5^/well) a day before transfection with a pCG1-Spike/PEI mixture. The next day, the cells were infected with the VSVΔG*/G virus in a serum-free medium for 1 h, after which the medium was removed and the cells were washed with PBS before the complete medium supplemented with an anti-VSV-G antibody (8G5F11, Kerafast, Boston, MA, USA) added to the cells. After 18 h, the cell supernatant was centrifuged, and the cleared pseudovirus supernatant was aliquoted and stored at −80 °C until use. For the neutralization assay, HEK293 were seeded (2.5 × 10^4^/well) a day before transfection with plasmids encoding ACE2 (pCG1-ACE2, a kind gift from Stefan Pölhmann), pCMV3-C-Myc-TMPRSS2, and phRL-TK (encoding Renilla luciferase). Immunized mice sera were pre-incubated with the spike pseudovirus for 30 min before addition to the cells. The next day, the medium was removed, and the cells were lysed in a passive lysis buffer (Biotium, Fremont, CA, USA). Luciferin substrate (Xenogen, PerkinElmer, Waltham, MA, USA) was used to detect firefly luciferase activity as a measure of pseudovirus infection and coelenterazine H (Xenogen) to follow the Renilla luciferase activity for the determination of transfection efficiency and normalization.

### 2.15. Neutralization Assay Based on Inhibition of ACE2 Spike Interaction

Recombinant human ACE2-Fc (Genscript, Piscataway, NJ, USA) at a concentration of 2 μg/mL in a phosphate buffer saline (PBS) was adsorbed to wells of ELISA plates at 4 °C overnight. Serum dilutions were prepared in 1% BSA/PBS-T and incubated with equal amounts of spike protein (the final concentration of the spike protein in all samples was 0.1 μg/mL) for 1 h at 37 °C. After blocking in 1% BSA/PBS-T for 1 h at 37 °C, the plates were washed in PBS-T, and pre-incubated serum-spike samples were added to the wells and incubated for 2 h at room temperature. After washing, the plates were incubated with an HRP conjugated engineered form of streptavidin (streptactin-HRP) (1:) in 1% BSA/PBS-T for 1 h at room temperature. After the final wash, a TMB substrate was added, and the reaction was stopped by the addition of an acid solution (3M H_3_PO_4_). Absorbance was measured by the Synergy Mx microtiter plate reader (Biotek, Winooski, VT, USA).

### 2.16. T-cell response on Mouse Splenocytes

To determine the presence of antigen-specific cytotoxic CD8a+ T cells, mice spleens from RBD DNA or RBD-scaffold DNA immunized animals were harvested. Single cell suspensions from spleens without enzymatic treatment were obtained using the tissue dissociator gentleMACS^TM^ Dissociator, according to the manufacturer’s instructions (Miltenyi Biotec, Bergisch Gladbach, Germany). CD8^+^ T cells from spleen cell suspension were isolated using mouse CD8a^+^ T Cell Isolation Kit (Miltenyi Biotec; 130-104-075, Miltenyi Biotec, Bergisch Gladbach, Germany), according to the manufacturer’s instructions. Cells were isolated based on negative selection using LS columns, obtaining up to 10^8^ labeled cells. To determine RBD-specific cytotoxicity, mouse NIH-3T3 cells were seeded into 24-well plates (1 × 10^5^/well); the next day, the cells were transfected with pCG1-hACE2 (900 ng/well) and pCMV-TMPRSS2 (30 ng/well) plasmids. The following day, cells were infected with spike pseudovirus with a bioluminescent reporter. The day after, isolated CD8a + T cells (1 × 10^5^/well) in RPMI1640 cell medium were added. After 24 h, bioluminescence was determined using IVISIII (Perkin Elmer, Waltham, MA, USA) after the addition of D-luciferin (500 μM), showing the state of the RBD-specific cytotoxicity of the CD8 + T cells isolated from RBD DNA- or RBD-scaffold DNA-vaccinated animals. Bioluminescence values are presented as average radiance (p/s/cm^2^/sr), which were determined using Living Image^®^ software. From the average radiance values (ARV), the percentage of infected NIH-3T3-specific lysis was calculated using the following formula: % specific lysis = 100 × (spontaneous death ARV-test ARV)/(spontaneous death ARV-maximal killing ARV).

To determine cell-specific response, mouse splenocytes were seeded into 24-well plates (1 × 10^6^/well). Cells were then stimulated with SARS-CoV-2 (spike glycoprotein) PEP-LIPS-Pool peptide pool (peptides&elephants GmbH; LB01792), consisting of 316 peptides at a final concentration of 10 μg/mL. The next day, mouse IFNγ was determined using the mouse IFN gamma ELISPOT ELISA Ready-SET-Go!™ Kit; (15531137, eBioscience, San Diego, CA, USA) according to the manufacturer’s instructions. *CxCl9* mRNA fold difference was determined using quantitative PCR. The mRNA was isolated by using the High Pure RNA Isolation Kit (Roche) according to the manufacturer’s protocol. A total of 200 ng of mRNA was transcribed with the High Capacity cDNA Transcription Kit (AB Applied Biosystems). qPCR was performed using the LightCycler^®^ 480 SYBR Green I Master mix (Roche, Basel, Switzerland) on the LightCycler 480 instrument (Roche, Basel, Switzerland), where the following primers were used: CxCl9-forward 5′- *cctagtgataaggaatgcacgatg*-3′, CxCl9-reverse 5′- *ctaggcaggtttgatctccgttc*-3′, GAPDH-forward 5′-*aaggccggggcccacttgaa*-3′, and GAPDH-reverse 5′- *tgggggcatcggcagaaggg*-3′.

### 2.17. Lymph Node Trafficking

The RBD and RBD-ban were labeled with the fluorescent dye Alexa Fluor647 (AF647), according to the manufacture’s protocol (AlexaFluor 647 conjugation kit lightning link, Abcam, ab269823). In brief, the modifier reagent (100 μL) was added to a solution of protein (1 mL, 1 mg/mL PBS). Afterward, the solution was mixed with a lyophilized AlexaFluor 647 conjugation mix. After 10 min of incubation at 25 °C, the reaction was terminated with the addition of the quencher reagent (100 μL). The labeled protein was desalted using a PD-10 column and was concentrated (300 μL, approx. 2 mg/mL PBS). A total of 50 μg of AF-647 the labeled protein was injected into the mouse foot pad (RBD-AF647 into the left and RBD-bann-AF647 into the right). Protein trafficking was observed using the in vivo imaging system IVIS^®^ Lumina Series III (Perkin Elmer, Waltham, MA, USA). The fluorescence signal that depicted the presence of the AF647-labeled protein was determined. Fluorescence was measured using AlexaFluor 647 Probe settings. The mice were exposed for 0.5–5 s using bining factor 8, f number 2. The data were analyzed with Living Image^®^ 4.5.2 (Perkin Elmer, Waltham, MA, USA), where the area surrounding the PLN was determined as the ROI. After a defined period of time, the mice were humanely sacrificed, and their hind limbs were removed to visualize the total fluorescence efficiency in the PLN.

### 2.18. Surrogate Assay of Protection of Viral Infection by Immunization

**Figure 1:**
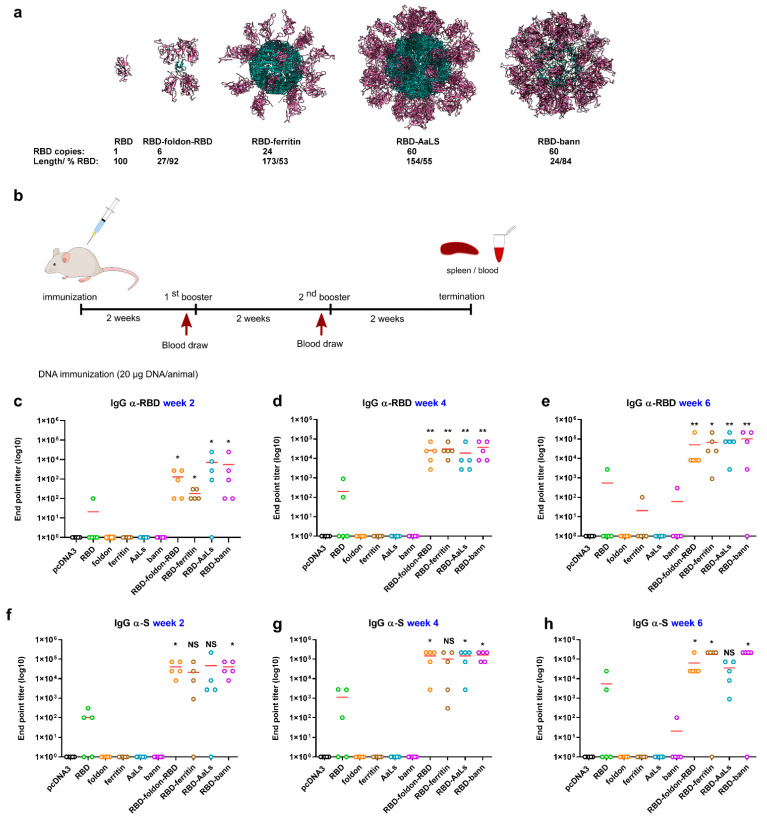
Scaffolded RBDs induce high titers of antibody production *in vivo.* Molecular models of RBD domains are shown in violet and scaffold cores in blue. The number of RBD domains per particle, length of the scaffolding domain, and fraction of amino acid residues of RBD in the assembly are listed below each model (**a**). Mice were immunized with plasmid DNA encoding RBD, scaffolded RBD variants, or scaffolds alone, according to the indicated immunization schedule (**b**). End point titer (EPT) values of total IgG against RBD (**c**–**e**) and against whole length spike protein (**f**–**h**) are shown. Graphs represent the mean EPTs of groups of mice (*n* = 5 per group). Each dot represents an individual animal. * *p* < 0.05; ** *p* < 0.01. All *p* values are from the Mann-Whitney test compared to RBD group.

Balb/c mice were immunized with RBD-bann according to the scheme presented in Figure 1. Two weeks after the boost, the mice were transfected by 30 μL of the plasmid mixture of jetPEI-in vivo and plasmid DNA (20 μg hACE2, 1 μg TMPRRS per animal) via intranasal administration in inhalation anesthesia. The next day, the mice were again intranasally infected with 70 μL of VSV-S pseudovirus. Twenty-four hours later, the mice received 150 mg/kg of their body weight of D-luciferin (Xenogen) intraperitoneally and were in vivo imaged by using IVIS^®^ Lumina Series III (Perkin Elmer, Waltham, MA, USA). The bioluminescence that depicted the state of the pseudovirus infection was determined. The data were analyzed with Living Image^®^ 4.5.2 (Perkin Elmer, Waltham, MA, USA).

### 2.19. Reporter Virus Strain, Stock Preparation, Plaque Assay, and In Vitro Infection

SARS-CoV-2-GFP (PMID: 32365353) was produced by infecting Vero E6 cells cultured in DMEM medium (10% FCS, 100 μg/mL Streptomycin, 100 IU/mL Penicillin) for two days at MOI 0.01. Viral stock was harvested and spun twice (1000 g/10 min) before storage at −80 °C. The titer of the viral stock was determined by a plaque assay. Confluent monolayers of Vero E6 cells were infected with serial five-fold dilutions of virus supernatants for 1 h at 37 °C. The inoculum was removed and replaced with serum-free MEM (Gibco, Life Technologies) containing 0.5% carboxymethylcellulose (Sigma-Aldrich, St. Louis, MO, USA). Two days post-infection, the cells were fixed for 20 min at room temperature with formaldehyde directly added to the medium to a final concentration of 5%. The fixed cells were washed extensively with PBS before staining with H_2_O containing 1% crystal violet and 10% ethanol for 20 min. After rinsing with PBS, the number of plaques was counted, and the virus titer was calculated.

### 2.20. Serum Virus Neutralization Assay (SARS-CoV-2-GFP)

Vero E6 cells were seeded into 96-well plates at a density of 10,000 cells per well in a DMEM medium (10% FCS, 100 ug/mL Streptomycin, 100 IU/mL Penicillin) one day before the assay. A virus inoculum, corresponding to an MOI 1, was incubated with diluted mice serum for 1 h at room temperature (total volume of 100 μL per well) before being transferred to the cells. The inoculum was replaced with a fresh medium 1 h later, and the cells were incubated for 36 h at standard culturing conditions before the confluency and GFP intensity were measured using an IncuCyte S3 Live Cell Analysis System (4× magnification, one image per well, phase and GFP channel). The normalized GFP intensity was computed using the IncuCyte S3 software (Essen Bioscience; version 2019B Rev2, Sartorius, Göttingen, Germany) as integrated GFP intensity per well divided by the total area of the cells per well. The analysis and visualization were performed in R (4.0.2).

## 3. Results

### 3.1. Design of RBD-Presenting Polypeptide Nanoparticle Scaffolds of Different Size and Oligomerization State

The trimeric S protein has been used in most vaccine candidates. Additionally, a dimeric and trimeric RBD domain demonstrated increased immunogenicity [2,43] in comparison to the monomeric RBD. Diverse platforms have been used to generate nanostructured vaccines, such as ferritin-scaffolded vaccines against influenza, HIV-1, and RSV [24,44]. While such strategies are attractive, an insistent concern remains the immunogenicity of the scaffold domain. Aiming to explore the effect of the type of a nanoscaffold and minimize the size of the scaffold in order to reduce the antibody response against the scaffold, several variants have been engineered, aiming to present one, six, 24, and 60 copies of RBD by genetically fusing it to the foldon (RBD-foldon-RBD), ferritin (RBD-ferritin), lumazine synthase (RBD-AaLs), and β-annulus peptide (RBD-bann). These scaffolds range in size from 24 to 173 amino acid residues, representing 7–46% of the residues in the genetically engineered fusion constructs. The stoichiometry of six was achieved in the RBD-foldon-RBD construct, where the RBD domain was fused to both the amino- and carboxy-terminus of the trimerizing foldon domain [45]. Since the RDB is much larger than the foldon peptide, it can, according to the molecular model (Figure 1a), more efficiently shield the foldon trimerization core in comparison to the previously used fusion at only a single terminus [5]. Higher antigen stoichiometries can mask the scaffolding domain even more efficiently. Ferritin forms a 24-meric cage and has been frequently used for the presentation of polypeptide antigens, as it can present eight trimers on each particle [24,25,44]. However, the density of RBD domains at its surface is lower than for the other designed assemblies (Figure 1a). An even larger number of RBD domains is predicted to be presented on the icosahedral *Aequifex aeolicus* lumazine synthase (AaLs), which has been used before for nanostructured vaccines [39,46]. A short peptide, comprising 24 amino acid residues based on the viral β-annulus protein from the tomato bushy stunt virus, has been shown to spontaneously form nanoparticles approximately 40 nm in diameter [32] and has been used before to attach different small cargo molecules, such as DNA or peptides [47,48,49]. We hypothesized that this peptide could be fused to a large protein antigen domain to generate soluble assemblies for vaccination. The molecular model was constructed according to the icosahedral packing of its parent viral capsid, which comprises 60 copies.

Nanostructuring of the antigen was combined with the nucleic acid delivery to take advantage of the speed, cost of production, and stability of a vaccine. Constructs for all scaffolded RBDs were encoded in plasmids, including a signal sequence for in situ protein production and secretion, as cytoplasmic expression has a low immune response efficiency [50]. A plasmid DNA-based vaccine represents an attractive vaccine platform on account of cost-effective massive production and DNA plasmid stability, eliminating the need for cold chain transportation, which may limit the worldwide deployment of some vaccines. To confirm the stability, the plasmid DNA vaccine was incubated at room temperature, where no degradation or decreased protein production upon transfection of cells occurred after seven days, emphasizing an important advantage of a DNA vaccine platform (Figure S1). RBD and RBD fusion variants were expressed from plasmid DNA and secreted into supernatants of Expi293F cells, as shown by Western blot (Figure S2).

### 3.2. Scaffolded RBDs Induce a Robust Humoral Immune Response In Vivo

Mice were immunized with DNA plasmids encoding for RBD, scaffolded-RBD variants, or scaffolds alone. Two booster immunizations two weeks apart followed the initial immunization, with blood samples collected prior to each boost (Figure 1b). IgG antibody titers against RBD and spike proteins two weeks after the priming immunization were significantly higher for scaffolded RBD domains (Figure 1c–e). Titers against the Spike protein were two orders of magnitude higher in all scaffolded variants than in the case of RBD (Figure 1f–h). Following the first booster immunization, antibody titers were similar for all scaffolded RBD variants, increasing 95-, 130-, 150-, and 180-fold for RBD-AaLs, RBD-foldon-RBD, RBD-ferritin, and RBD-bann, respectively—all orders of magnitude higher in comparison to RBD (Figure 1d). Titers after the third immunization demonstrated that one boost is sufficient to reach the plateau response (Figure 1e).

**Figure 2:**
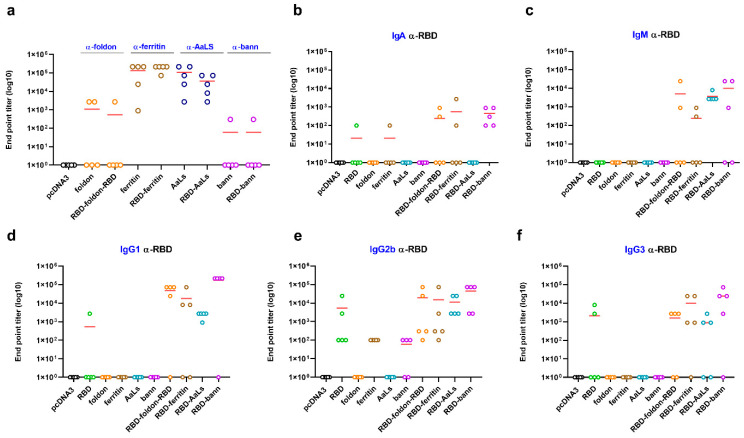
Antibody response depending on the nanoscaffolding domain and Th-1 biased antibody response in vivo. Mice were immunized with the indicated plasmid DNA coding for scaffolds or RBD variants, and end point titers of total IgGs against different scaffold molecules were measured with ELISA (**a**). End point titers of different isotypes (IgA (**b**) and IgM (**c**)) and subclasses (IgG1 (**d**), IgG2b (**e**), and IgG3 (**f**)) of immunoglobulins against RBD were determined by ELISA six weeks after the first immunization. Graphs represent the mean EPT of groups of mice (*n* = 5 per group). Each dot represents an individual animal.

All scaffolded RBD-domains led to production of IgG1, IgG2b, and IgG3 with lower titers of IgM and IgA and no IgG2a, indicating a Th1-biased response, typically observed for DNA and commonly elicited by viral pathogens (Figure 2b–f) [51]. While plasmids in combination with the jetPEI transfection reagent were used initially, further immunization experiments demonstrated that naked plasmid DNA produced comparable antibody titers compared to the complexed jetPEI-DNA mixture (Figure S3).

Since SARS-CoV-2 is a respiratory virus, immunization in the respiratory tract is likely to be most effective in preventing infection and disease. Therefore, in addition to the intramuscular injection of plasmids, intranasal and sublingual delivery of the plasmid vaccine was also tested. Intranasal immunization was at least equally as effective as intramuscular injection of the plasmid and may represent an attractive immunization strategy to prevent infection in the respiratory tract (Figure S4).

### 3.3. Immunogenic Properties of Scaffold Domains of the Nanoparticle Vaccines

Despite reports of vaccination with different types of scaffolds for the formation of nanoparticles, immune response against the scaffold has often been neglected, although immune response may be impaired in the case of repeated use of the same scaffolding or delivery platform [33,34]. Therefore, the scaffolding domain should ideally be small and hypoimmunogenic. In the case of the β-annulus and foldon peptides, the two smallest scaffolding domains tested in this study, the antibodies against the scaffolding were barely detectable, while larger lumazine-synthase and ferritin scaffolding domains exhibited a strong immunogenic response (Figure 2a). Immunogenicity of the foldon peptide has been reported when fused to a larger antigen [40] but was suppressed in the RBD-foldon-RBD construct, likely due to B cell epitope shielding. Hypo-immunogenicity of the β-annulus peptide may be due to its amino acid composition, and it represents a highly suitable scaffolding domain, which is, in this respect, superior to large scaffolds such as ferritin or lumazine synthase. A further decrease in the immune response against the scaffold could be achieved by a heterologous prime-boost combination of different scaffold-based antigens at each stage (β-annulus- and foldon-RBD). Results demonstrated that antibody titers against RBD were the same as in the homogeneous prime-boost regimens (Figure S5), while immune response against the scaffold was not observable or minimal (Figure S6).

### 3.4. Sera of Mice Immunized with a RBD-Bann Encoding Vaccine Neutralize Spike-ACE2 Interaction In Vitro

**Figure 3:**
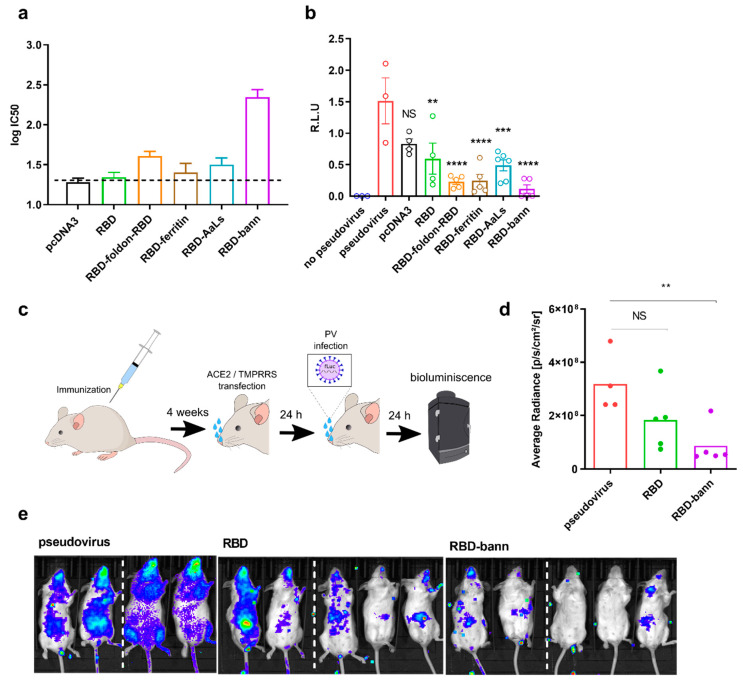
Neutralization of ACE2 binding and viral infection of RBD-scaffolded DNA vaccines. (**a**–**b**) Neutralization of the binding of the spike protein to the hACE2 receptor determined by an ELISA-type assay and inhibition of pseudoviral infection of cells by mouse antisera. Sera of mice immunized with DNA vaccines comprising different scaffolded RBDs were diluted and pre-incubated with a spike protein. Afterwards, Spike bound to ACE2 was detected using streptactin-HRP. The mean and SEM of six (RBD-AaLs) or five (all others) biological replicates are shown (**a**). Sera of mice immunized with DNA vaccines encoding scaffolded RBD were diluted 50-fold, and the spike-pseudotyped virus infection of ACE2 and TMPRSS2-transfected HEK293 cells was measured by luminescence. The mean and SEM of six (RBD-AaLs), five (RBD-bann, RBD-foldon-RBD, RBD-ferritin), or four (empty pcDNA3 vector, RBD) biological replicates are shown (**b**). ** *p* < 0.01, *** *p* < 0.001, **** *p* < 0.0001. All *p* values are from one-way ANOVA followed by Tukey’s multiple comparisons test. (**c**–**e**) Protection of infection by DNA plasmid immunization in a mouse model. Mice were immunized by two injections of different RBD plasmids separated by two weeks. After four weeks, hACE2 and TMPRRS were introduced by intranasal plasmid transfection followed by intranasal infection with SARS_CoV-2 S-typed virus (PV). Luminescence based on pseudovirus intranasal infection was measured after 24 h (**c**). Quantification of bioluminescence average radiance. Each dot represents an individual animal (pcDNA3 *n* = 4; RBD and RBD-bann *n* = 5). ** *p* < 0.01. All *p* values are from the Mann–Whitney test (**d**). Bioluminescence imaging revealing the protective state of immunized animals against pseudovirus infection in animals. The dashed line represents the merging of pictures of mice from the same test group taken separately (**e**).

The induction of neutralizing antibody production generally correlates with protective immunity. Neutralization of the engagement of the ACE2 receptor by the viral spike protein was investigated by an ELISA competition assay, which has been shown before to correlate well with neutralization of viral infection [52]. Results showed the highest neutralization by the sera of mice immunized with the RBD-bann encoding vaccine with an IC50 dilution of ~1:220 (Figure 3a and Figure S7) and weakest by sera of RBD DNA vaccine immunized mice. Viral neutralization was additionally tested using an S-pseudotyped VSV viral assay (Figure 3b). Similar to the binding neutralization assay, the RBD-bann encoding vaccine preformed best in terms of neutralizing infection of host cells, followed by RBD-foldon-RBD, RBD-ferritin, and RBD-AaLs. In both assays, protection was significantly lower in mice immunized with DNA encoding RBD and was above the protection level established in nonhuman primates [53].

### 3.5. Immunization with RBD-bann Is Protective In Vivo

To test vaccines in mice, hACE2 must be provided to sensitize mice to SARS-CoV-2 infection. hACE2-transgenic mice have been generated [54]; however, their availability was not sufficient for all interested researchers; therefore, the hACE2 receptor has also been introduced through AAV delivery [55]. Here, we developed an infection model where plasmids encoding hACE2 and TMPRRS were introduced into mice by intranasal transfection, a technique that could be rapidly adapted to the selected viral infection models. Indeed, intranasal infection of hACE2/TMPRRS-transduced mice with SARS-CoV-2 spike pseudotyped luciferase expressing replication-incompetent virus led to accumulation of luciferase activity in contrast to non-transduced control mice. Although the pseudotyped virus does not replicate, it nevertheless represents a convenient system by which to recapitulate virus neutralization efficiency in the upper respiratory tract of immunized mice, since neutralizing antibodies target viral recognition of the cellular receptor. Moreover, this assay does not require a biosafety level 3 animal facility, making it safer and faster to use by a wide range of researchers. Mice were immunized with RBD, RBD-bann, or a control vector and challenged with VSV-S(CoV-2) four weeks later. Notably, strong protection against pseudoviral infection was observed in mice immunized with RBD-bann and weaker protection in mice immunized with RBD as compared to control mice (Figure 3c–e). This indicates that immunization with the nanoscaffolded RBD allows efficient protection against intranasal infection with a virus recapitulating the early stages of SARS-CoV-2 infection in vivo.

### 3.6. Immunization with RBD-Bann Plasmid Induces Spike Protein-Specific Cytotoxic T Cells

**Figure 4:**
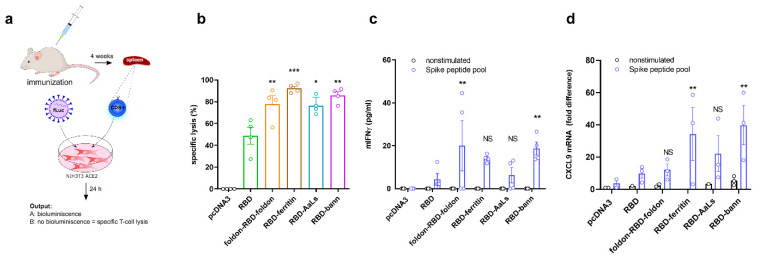
Spike protein-specific cytotoxic killing by lymphocytes from immunized animals. Cytotoxic T-cell killing from immunized mice against cells expressing viral S protein. Mice were immunized with different combinations of RBD plasmid DNA, and mice spleens were harvested after four weeks. The cytotoxic effect of isolated CD8^+^ T cells was determined on pseudovirus-infected hACE2 and TMPRRS-transfected NIH-3T3. Bioluminescence was determined 24 h post co-culture of murine spleen cells with NIH-3T3 cells (**a**). Based on radiance values obtained from pseudovirus infection, specific lysis of NIH-3T3 cells was calculated (**b**). Mouse splenocytes from DNA-immunized animals were stimulated with a spike peptide pool, IFNγ was measured 24 h post-stimulation (**c**), and *CxCl9* mRNA fold expression was determined (**d**). Each dot represents spleen cells from an individual animal (*n* = 4). * *p* < 0.05, ** *p* < 0.01, *** *p* < 0.001. All *p* values are from one-way ANOVA followed by Tukey’s multiple comparisons test.

Since the RBD domain also contains several T-cell epitopes, it was expected to trigger both a CD4 and a CD8 T-cell response. CD8 cells were isolated from mice spleens harvested four weeks after the initial immunization with either DNA plasmid coding for RBD or scaffolded RBD (foldon-RBD-foldon, RBD-ferritin, RBD-AaLs, and RBD-bann). Strong specific augmentation of cell killing and IFNγ production was observed upon coculturing isolated CD8 T cells with infected NIH-3T3 cells, primarily in the case of mice immunized with scaffolded RBD vaccines (Figure 4a,b and Figure S8). Furthermore, stimulation of splenocytes from DNA-immunized mice with a spike peptide pool led to the highest T-cell cytokine production in RBD-bann and foldon-RBD-foldon vaccinated mice (Figure 4c,d). Together, these results suggest that this type of vaccine triggers a strong T-cell response, which has been shown to play a role in protection [53].

### 3.7. Immunization with RBD-Bann Protein Induces Potent Antibody Response

**Figure 5:**
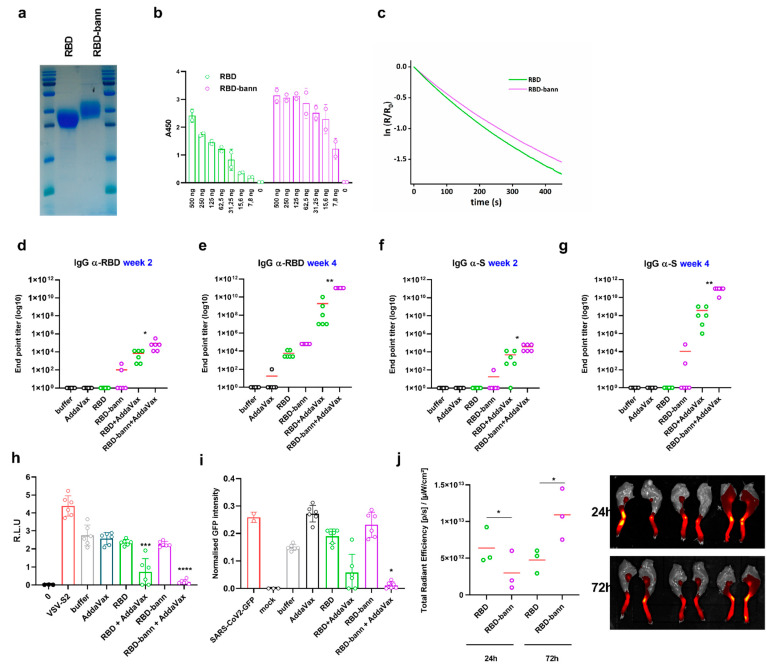
Augmented antibody titer, viral neutralization, and lymph node retention of RBD-bann protein vaccine. RBD variants were isolated from the supernatants of mammalian cells via affinity chromatography and analyzed for purity and specificity via SDS-PAGE (**a**) and the binding of recombinant proteins at a serial dilution, or buffer alone (0), to immobilized human ACE2 receptor was measured in an ELISA assay (**b**). Dissociation of RBD and RBD-bann from immobilized hACE2 was measured by SPR (**c**). Mice were immunized with RBD and RBD-bann proteins purified from mammalian cell supernatant with/without squalene-mediated adjuvant. End point titer (EPT) for total IgG against RBD (**d**–**e**) and against spike protein (**f**–**g**). Graphs represent the mean EPTs of groups of mice (*n* = 6 per group). Each dot represents an individual animal. * *p* < 0.05; ** *p* < 0.01. All *p* values are from the Mann–Whitney test compared to RBD group + AddaVax. Neutralization titer in mice sera was determined using a pseudovirus system. Sera of mice immunized with isolated proteins were diluted 50-fold, and Spike-pseudotyped virus infection of ACE2 and TMPRSS2-transfected HEK293 cells was followed by luminescence. Mean and SEM biological replicates are shown (**b**). * *p* < 0.05, *** *p* < 0.001, **** *p* < 0.0001. All *p* values are from one-way ANOVA followed by Tukey’s multiple comparisons test compared to the buffer group (**h**). A serum virus neutralization assay was performed by infecting Vero E6 cells with SARS-CoV-2-GFP reporter virus (MOI 1) with prior incubation of inoculum with dilutions of mouse sera (1:50) immunized with indicated isolated proteins. As control cells were left uninfected (mock), GFP signal was measured 36-h post-infection using the Incucyte S3 live-cell imaging system. Each point represents the mean of two technical replicates, and the mean +/− SD from six mice per group is indicated. Indicated *p*-values apply for comparison between individual conditions and the dilution-matched buffer condition and were calculated using a one-sided Student’s *t*-test between RBD + Addaax and RBD-bann + Addavax. * *p* < 0.05 (**i**). Trafficking of labeled isolated proteins into the popliteal lymph nodes (PLN). AF-647 labeled RBD proteins were injected into the foot pad (RBD left, RBD-bann right). The fluorescence signal of AF-647 was determined in the popliteal lymph node, depicting the presence of labeled RBD. Each dot represents the measurement of the region of interest (ROI) of an individual animal PLN. * *p* < 0.05 derived from two-tailed paired *t*-test (**j**).

In the context of plasmid vaccine application, RBD-bann exhibited the best immunological properties. To demonstrate that the fusion of RBD with the β-annulus peptide can be functional in different vaccine platforms and that the observed effect was not due to different expression levels of variants in the context of plasmid DNA vaccines, RBD and RBD-bann proteins were produced, purified from mammalian cells (Figure 5a), analyzed in vitro by biochemical methods, and used for immunization of mice (Figure S9). The oligomerization and binding of isolated protein variants to ACE2 was analyzed by an ELISA assay (Figure 5b), surface plasmon resonance (SPR) (Figure 5c), dynamic light scattering (DLS) (Figure S10A,B), and SEC-MALS (Figure S10A). Interestingly, the RBD-bann protein was observed predominantly in the monomeric form in vitro, as the size of RBD and RBD-bann particles measured by DLS and SEC-MALS appears similar (Figure S10). Nevertheless, it exhibited slightly stronger binding on ELISA and a small decrease of the k_off_ value determined by SPR, which may be attributed to an equilibrium between the monomeric and oligomeric states. The proportion of oligomeric species may, however, differ in the in vivo milieu under the conditions of molecular crowding and weakly interacting biopolymers.

Immunization with isolated proteins in the presence of an adjuvant triggered a substantially stronger immune response than immunization with plasmids, similarly as reported before for the spike protein [43] (Figure 5d–g). Results of protein immunizations further demonstrate that antibody titers were substantially higher in mice immunized with RBD-bann in comparison to RBD (Figure 5d–g) as well as pseuodovirus neutralization (Figure 5h). To corroborate these results with infection, a competent SARS-CoV-2 virus was used in a virus neutralization assay employing serial serum dilution. In short, virus inoculum was incubated with dilutions of sera obtained from immunized mice and subsequently transferred to Vero E6 cells. GFP signal intensity, originating from the reporter virus, was measured 36 h post-infection. Cell confluence upon measurement was comparable for all tested conditions, indicating that different sera concentrations did not influence cell growth (Figure S11). We observed strong and specific virus inhibition when using sera derived from mice immunized with RBD and RBD-bann proteins in conjunction with an adjuvant, wherein the latter conferred significantly higher levels of neutralization (Figure 5i).

Since in vitro analysis indicated small but consistent physicochemical differences between RBD and RBD-bann, we analyzed trafficking of the two protein forms to lymph nodes in mice, as this might explain the difference in their immunogenic properties. It has been shown before that particulate antigens are transported more slowly to lymph nodes, yet their retention in lymph nodes was extended [20]. Indeed, we found that monomeric RBD was transported to lymph nodes faster than RBD-bann and reached a higher concentration 24 h after application; however, after 72 h, the amount of RBD-bann in lymph nodes was significantly higher than RBD, which at that time had already started to decrease (Figure 5j). This suggests that trafficking and maintenance of the β-annulus-fused RBD to lymph nodes differs from trafficking of RBD and contributes to higher potency of the immune response to the β-annulus-fused antigen.

## 4. Discussion

Several recent reports on SARS-CoV-2 vaccines using RBD have revealed high neutralization and protection against viral infection, where the response was substantially stronger for dimeric compared to monomeric RBD [5,43,55]. Here, four different types of genetically encoded scaffolds fused to RBD provided information on the response against the antigen and scaffolding domain. We demonstrate that a strong increase in antibody response was generated already by the hexamerization scaffold. The antibody titers in this study were comparable to several other vaccines that have advanced to clinical trials [2,3,5,7] and fulfill the correlates of protection as recently established in nonhuman primates [53]. The size and the degree of multimerization was not the only important factor for the observed level of neutralization, as the RBD-AaLs variant, which presents 60 copies of the antigen, was less efficient in comparison to six copies in RBD-foldon-RBD, which may be at least in part due to less-efficient self-assembly in human cells. Scaffolded antigen presentation generated a robust T-cell response, which supported the generation of neutralizing antibodies and the licensing of cytotoxic lymphocytes, which play a role in vaccine efficiency [53]. This demonstrates that the RBD domain implemented in nanostructured vaccines is effective as a source of T-cell epitopes. Significant attention has previously been devoted to the presentation of antigens on different scaffolds in a precisely defined geometry [30,56]; however, the results of RBD fusion with the β-annulus peptide, which bestowed the best immunogenic characteristics while apparently generating a low amount of oligomers in vitro, suggest that the precise spatial positioning of antigens is likely not required except for the generation of a desired natural-like arrangement of epitopes, such as trimerization. Insoluble aggregates based on amyloid fibril-forming peptides as vaccine scaffolds [57,58] are less efficiently transported to the lymph nodes and germinal centers [20,59]. Analysis of T-cell epitopes did not reveal any epitopes within the β-annulus peptide. An explanation for the high efficiency of the β-annulus-scaffolded vaccine may be a dynamic equilibrium between the oligomeric states of the assembly, which may facilitate trafficking and presentation to B-lymphocytes or its interaction with antigen-presenting cells. This explanation is supported by the observed longer retention of the RBD-bann antigen in lymph nodes.

Small scaffolds have the advantage of maximizing the fraction of the desired antigen in the fusion protein and are hypoimmunogenic, avoiding a nonproductive or potentially even disruptive diversion of the antibody response. While foldon has been reported as strongly immunogenic in the context of fusion to other proteins [40], its positioning at the core of the RBD-foldon-RBD assembly likely provides shielding, which decreased its immunogenicity. The β-annulus peptide contains predominantly weakly immunogenic residues, and our results demonstrated that the β-annulus peptide alone triggered a weak immune response. Immunization with AaLs- and ferritin-scaffolded RBD, in contrast, generated a strong response against the scaffold. A strong immune response against RBD-ferritin has been confirmed in a recent paper [55] submitted after the version of this report had been deposited to the bioRxiv [60]. This scaffold-targeted response could be deleterious for repeated immunizations, similarly to adenoviral vaccines [61]. The mouse infection model based on the intranasal transfection of the appropriate receptor in combination with pseudoviral infection as presented here enables fast adaptation of the infection protection model and its wide applicability without BSL3 lab requirement.

Here, protein antigen design using nanoscaffolding was combined with the nucleic acid delivery for the in situ production of nanoscaffolded antigens. Plasmid DNA is attractive as a vaccination platform due to its easy adaptability and stability as well as its cost of production, which may contribute to the worldwide accessibility of the vaccines, where it could facilitate vaccine logistics to regions without cold chain availability. The results presented here demonstrate that intranasal vaccination can trigger a high antibody response. DNA vaccines have also demonstrated efficiency as priming agents in protein booster combinations [62,63]; therefore, they could be used in combination with other vaccine platforms. Further, prime–boost combinations that involve DNA vaccines have been shown to boost mucosal immunity [63,64,65], which could represent strong therapeutic advantages for respiratory viruses.

We demonstrated that the β-annulus RBD fusion improved immune response not just in the form of a DNA vaccine, but also as an isolated protein vaccine and likely also for other vaccine platforms such as mRNA or vector vaccines. Nevertheless, the general applicability of the β-annulus genetic fusion strategy will have to be tested for other protein antigens.

## 5. Conclusions

In conclusion, the genetic fusion of RBD to several scaffold domains elevated immune responses in mice when administered as a plasmid DNA-based vaccine, wherein fusion to the beta annulus peptide resulted in high levels of neutralizing antibody production and a robust T cell response. Fusion of target antigens to a small scaffolding domain enables fast design and implementation as nucleic acid-based vaccines, particularly in cases of epidemic emergencies. The genetic fusion of the RBD as presented here represents a promising therapeutic candidate and a promising platform for vaccines against other emerging viral diseases.

## Data Availability

All data needed to evaluate the conclusions in the paper are already included and/or available within the Supplementary Materials. Additional data related to this paper may be requested from the authors.

## Supplementary Materials

The following are available online at https://www.mdpi.com/article/10.3390/vaccines9050431/s1, Figure S1: Stability of the RBD-bann encoding plasmid, Figure S2. Production of RBD and scaffolded RBD variants in mammalian cells, Figure S3. DLS measurements of isolated proteins, Figure S4. DNA plasmid immunization with naked DNA, Figure S5. Immune response of different vaccine delivery routes in vivo, Figure S6. Total IgG in mice following switch immunization, Figure S7. Total IgG against scaffold in mice following switch immunization, Figure S8. Neutralization assay based on inhibition of ACE2 Spike interaction in the presence of sera of DNA immunized mice, Figure S9. Bioluminscence measurements of pseuodvirus infection of hACE2 and TMPRRS transfected NIH-3T3 after the addition of CD8^+^ cells isolated from mouse spleens, Figure S10. SDS-PAGE and Western blot of recombinant proteins isolated from mammalian cell supernatants, used in mice immunization, Figure S11. Vero E6 cell confluence in virus neutralization assay.
