# Supplementary material for: A Nanoscaffolded Spike-RBD Vaccine Provides Protection against SARS-CoV-2 with Minimal Anti-Scaffold Response
Source: Vaccines (Basel). 2021 Apr 27;9(5):431. doi: 10.3390/vaccines9050431 (PMC8146944; doi:10.3390/vaccines9050431)
Supplement: Supplementary file 1 [file vaccines-09-00431-s001.zip › vaccines-1176785-supplementary.pdf]

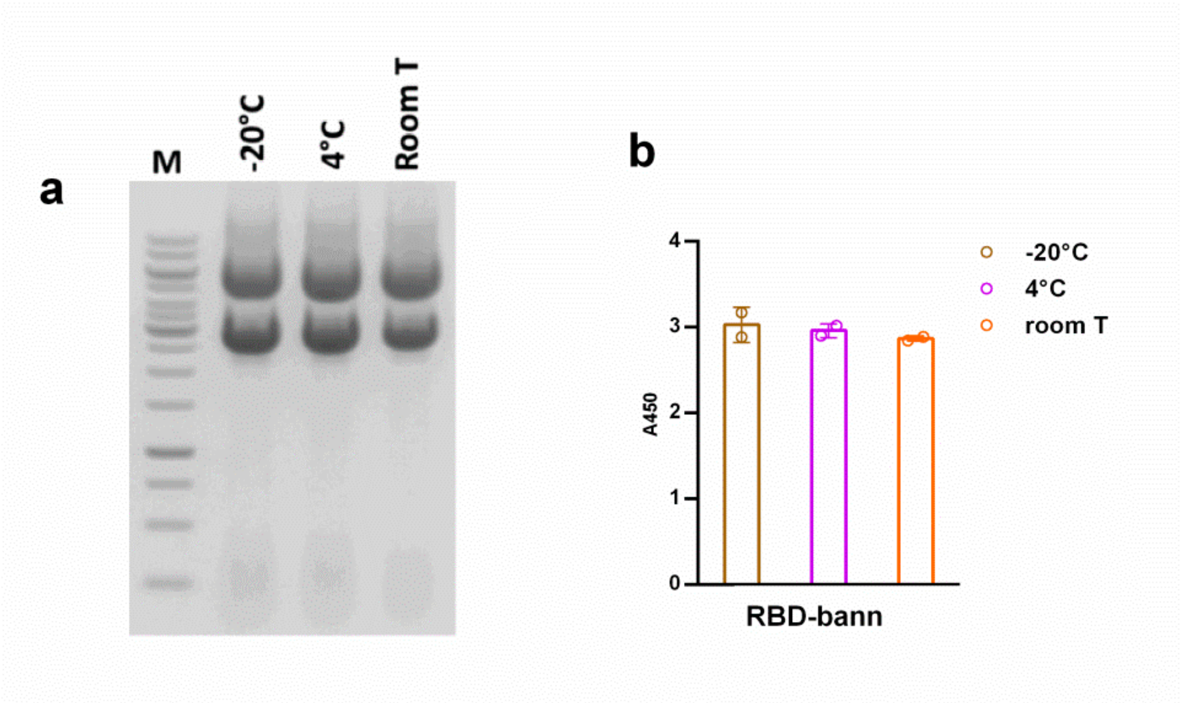

Figure S1. Stability of the RBD-bann encoding plasmid. The plasmid encoding RBD-bann was incubated for 1 week at −20°C, 4°C or at room temperature and analysed with agarose electrophoresis (A) or transfected into mammalian cells and expression of the RBD-bann protein was measured via sandwich ELISA in the cell supernatants (A).

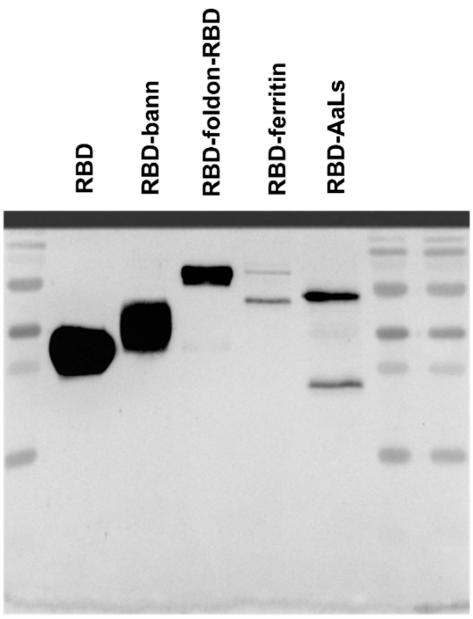

Figure S2. Production of RBD and scaffolded RBD variants in mammalian cells. Expi293F cells were transfected with constructs encoding RBD or scaffolded RBD variants and proteins were isolated from supernatants via affinity chromatography and analysed with Western blot.

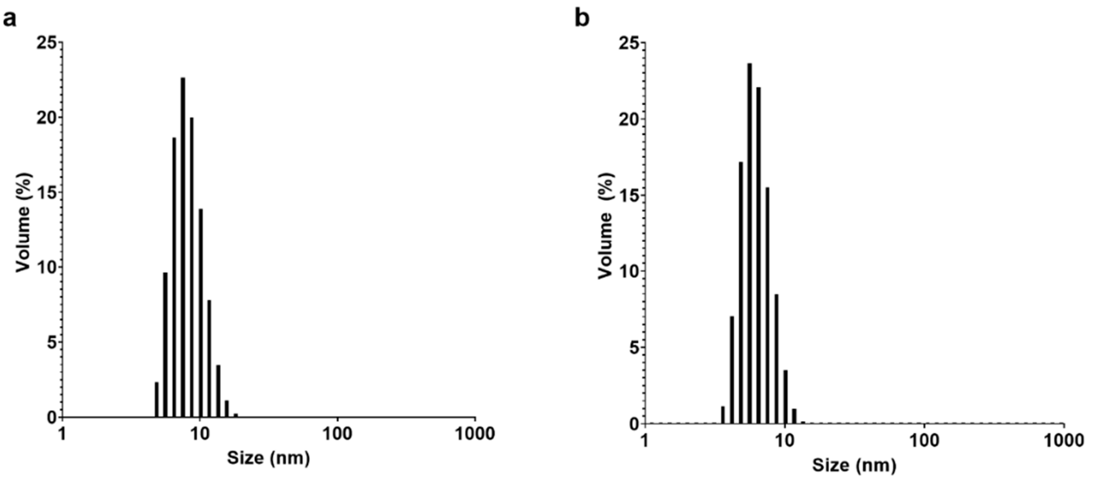

Figure S3. DLS measurements of isolated proteins. Expi293 isolated proteins were measured on DLS to determine its size; RBD (A) and RBD-bann (B).

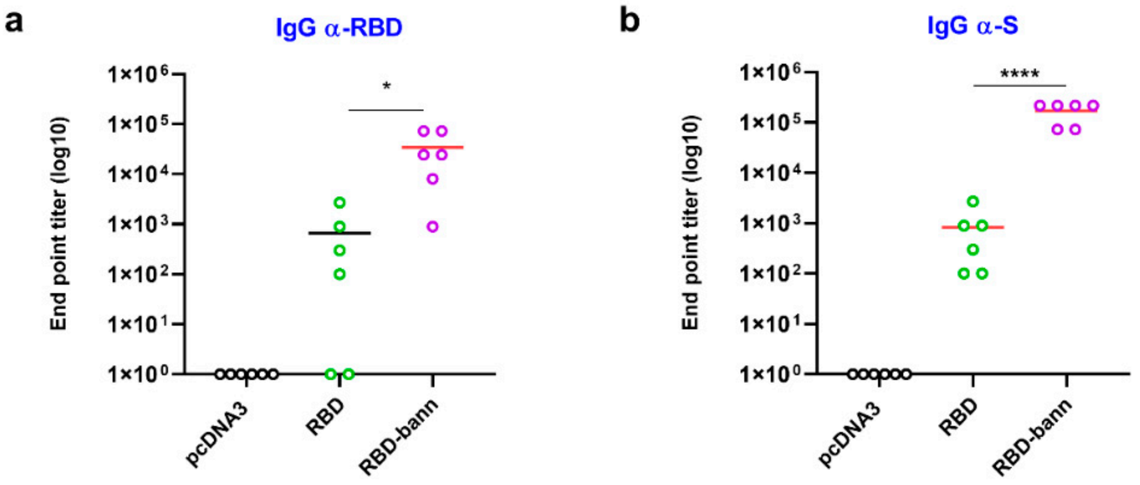

Figure S4. DNA plasmid immunization with naked DNA. Mice were immunized with 20 μg per animal of naked DNA (empty vector, RBD, RBD-bann), dissolved in 150 mM NaCl. End point titer (EPT) for total IgG against RBD (A) and against Spike protein (B) were determined by ELISA. Graphs represent mean of EPT of group of mice (n = 6 per group). Each dot represents an individual animal. *P < 0.05; ****P < 0.0001. All P values are from one-way ANOVA followed by Dunnett's multiple comparisons test.

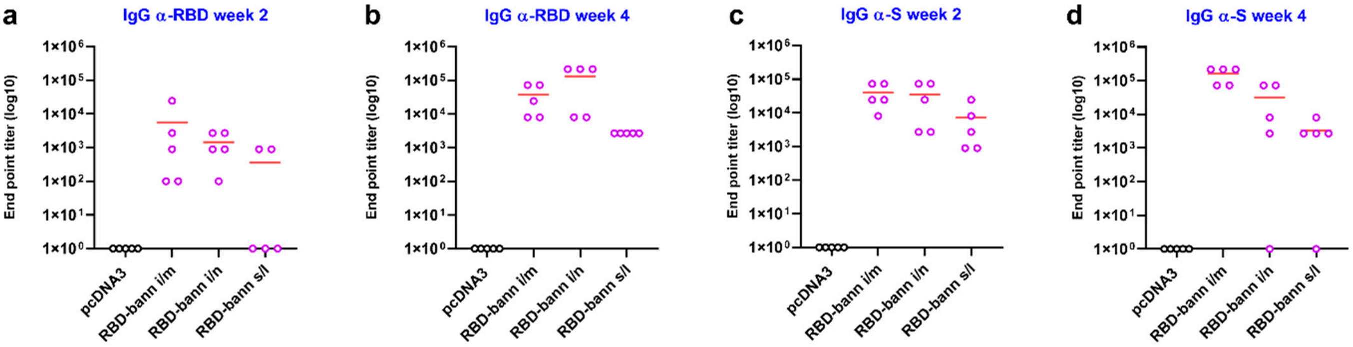

Figure S5. Immune response of different vaccine delivery routes *in vivo*. Mice were immunized with 20 μg per animal of naked DNA (empty vector, RBD-bann), dissolved in 150 mM NaCl via intramuscular (i/m), intranasal (i/n) or sublingual (s/l) route of immunization. End point titer (EPT) for total IgG against RBD (A, B) and against Spike protein (C,D) were determined by ELISA in mice sera. Graphs represent mean of EPT of group of mice (n = 6 per group). Each dot represents an individual animal.

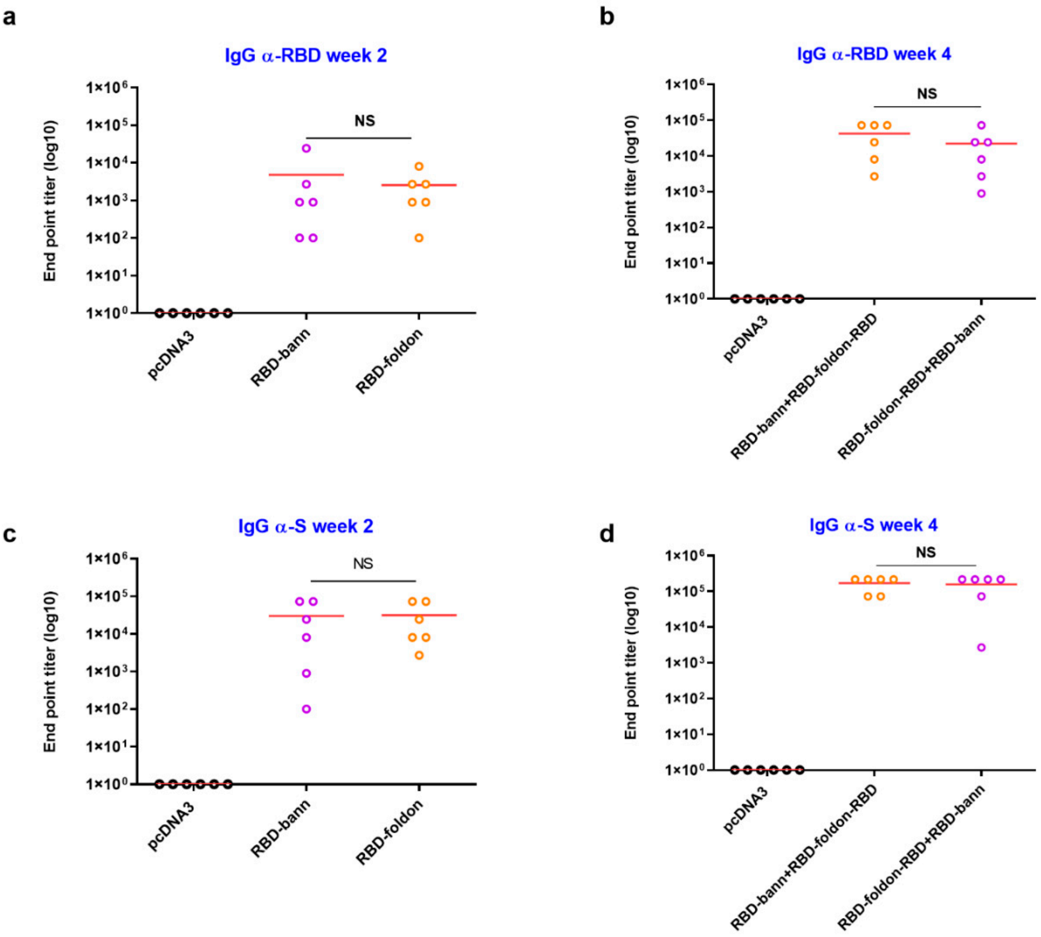

Figure S6. Total IgG in mice following switch immunization. Mice were immunized with differently scaffolded RBD plasmid DNA (β-annulus and foldon) for prime and boost immunization. Titers of antibodies against RBD following prime and boost (A,B) and against Spike protein (C, D) were determined via ELISA. Graphs represent mean of EPT of group of mice (n=6 per group). Each dot represents an individual animal. To determine NS, Mann-Whitney test was performed.

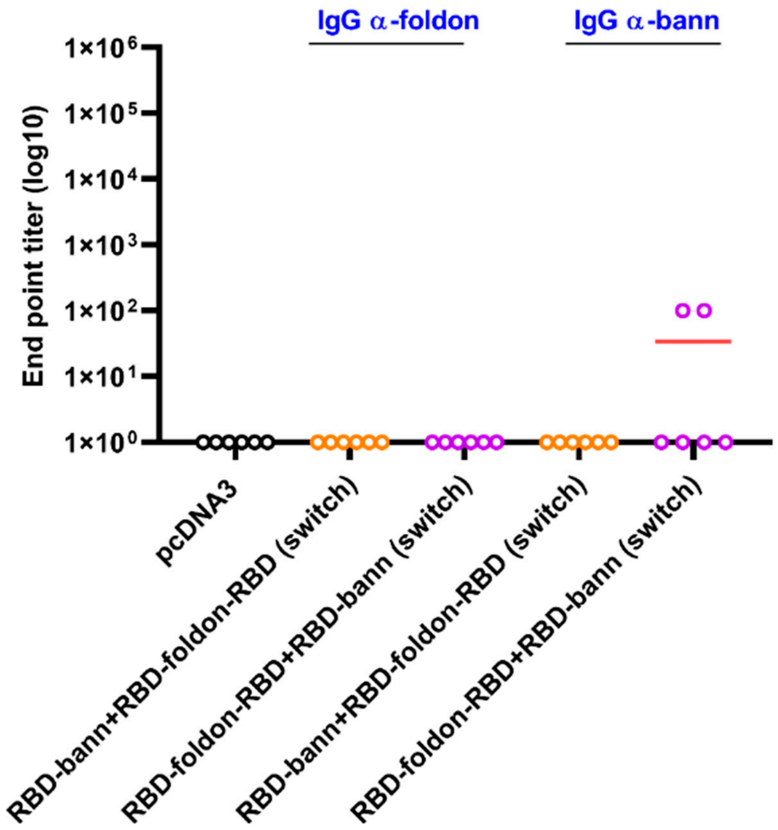

Figure S7. Total IgG against scaffold in mice following switch immunization. Mice were immunized with plasmid DNA encoding differently scaffolded RBD (β-annulus and foldon) for prime and boost immunization as indicated. Titers of antibodies against scaffold (depicted in blue) after prime and boost were determined via ELISA. Graphs represent mean of EPT of group of mice (*n* = 6 per group) after the boost. Each dot represents an individual animal.

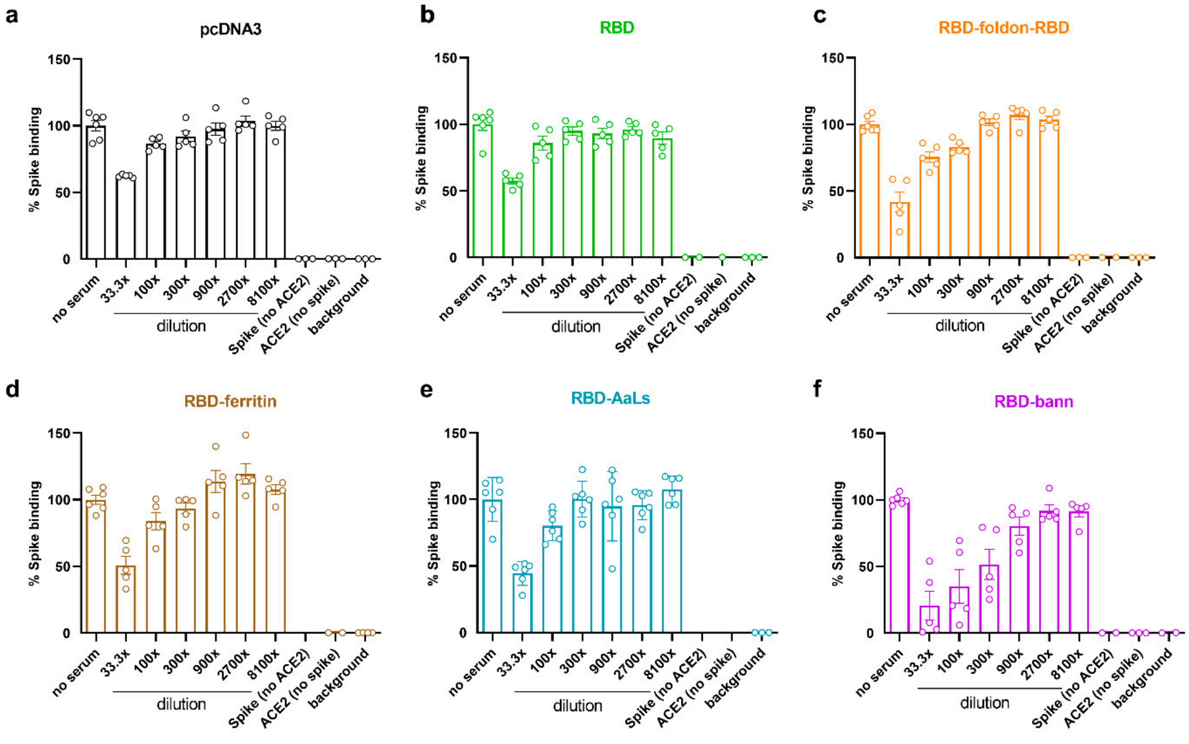

Figure S8. Neutralization assay based on inhibition of ACE2 Spike interaction in the presence of sera of DNA immunized mice. Sera dilutions of mice immunized with plasmid DNA encoding the different RBD scaffold variants or controls were preincubated with Spike protein. Inhibition of Spike and ACE2 protein interaction was determined via ELISA, indicating the neutralization effect of anti-Spike antibodies in sera of DNA immunized mice.

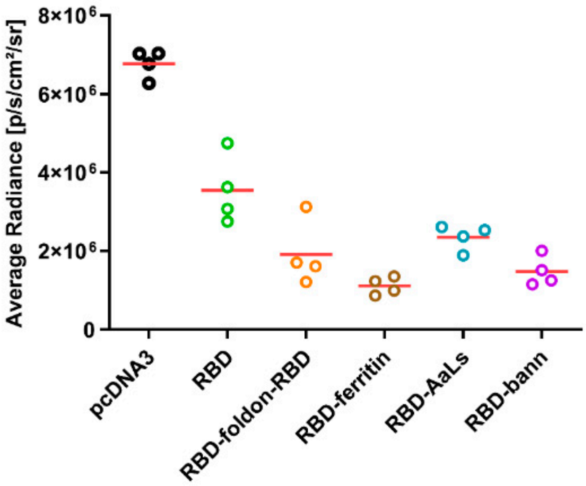

Figure S9. Bioluminscence measurements of pseudovirus infection of hACE2 and TMPRRS transfected NIH-3T3 after the addition of CD8^{+} cells isolated from mouse spleens. Twenty-four hours later bioluminscece was measured in cell coculture of mouse isolated CD8+ cells and NIH-3T3 cells, infected with pseudovirus.

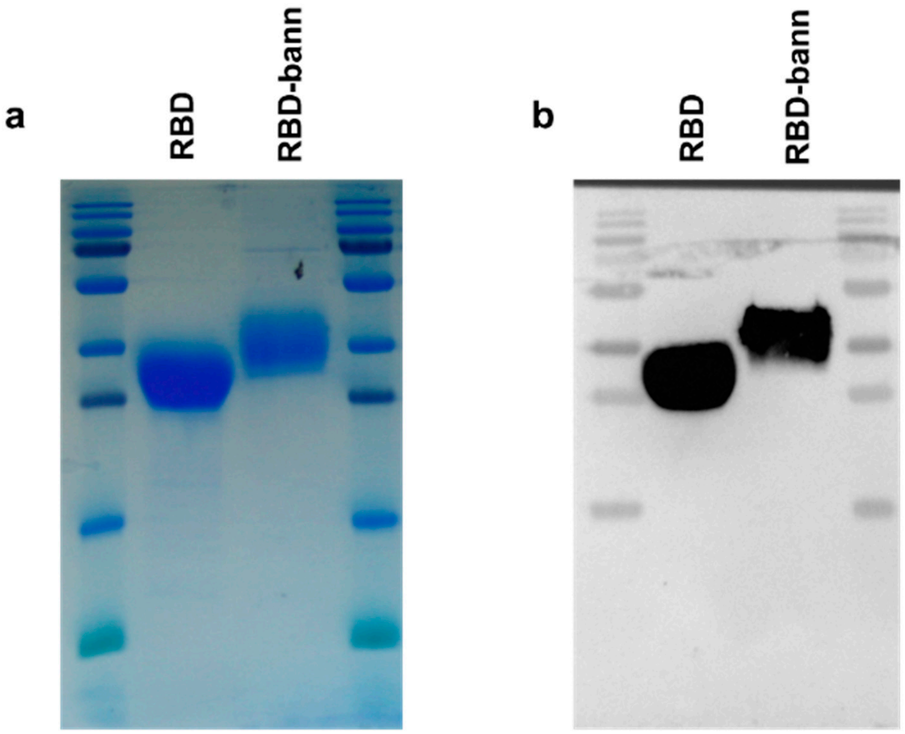

Figure S10. SDS-PAGE and Western blot of recombinant proteins isolated from mammalian cell supernatants, used in mice immunization. Expi293F cells were transfected with plasmids encoding RBD or RBD-bann and supernatants were harvested 5 days post transfection. Proteins were isolated via affinity chromatography and analysed for purity and specificity via SDS-PAGE and Western blot using anti-his antibodies.

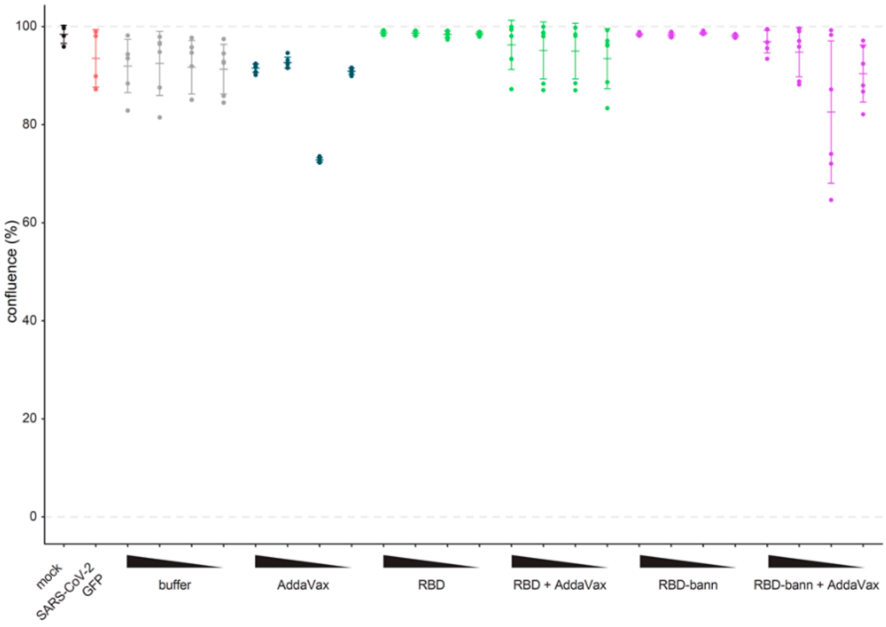

Figure S11. Vero E6 cell confluence in virus neutralization assay. Vero E6 cell confluence at the time of reporter virus signal acquisition in serum virus neutralisation assay was measured using Incucyte S3 live-cell imaging system. Each point represents mean of two technical replicates and mean +/- sd from 6 mice per group is indicated.
